# An alphavirus replicon-based vaccine expressing a stabilized Spike antigen induces protective immunity and prevents transmission of SARS-CoV-2 between cats

**DOI:** 10.1038/s41541-021-00390-9

**Published:** 2021-10-20

**Authors:** Martijn A. Langereis, Irina C. Albulescu, Judith Stammen-Vogelzangs, Morindy Lambregts, Ken Stachura, Suzan Miller, Angela M. Bosco-Lauth, Airn E. Hartwig, Stephanie M. Porter, Michelle Allen, Mark Mogler, Frank J. M. van Kuppeveld, Berend-Jan Bosch, Paul Vermeij, Ad de Groof, Richard A. Bowen, Randy Davis, Zach Xu, Ian Tarpey

**Affiliations:** 1 MSD Animal Health, Boxmeer, the Netherlands; 2 Division of Infectious Diseases and Immunology, Department of Biomolecular Health Sciences, Faculty of Veterinary Medicine, Utrecht University, Utrecht, the Netherlands; 3 Merck Animal Health, Elkhorn, NE USA; 4 College of Veterinary Medicine and Biomedical Sciences, Colorado State University, Fort Collins, CO USA; 5 Merck Animal Health, De Soto, KS USA; 6 Merck Animal Health, Ames, IA USA; 7 MSD Animal Health, Milton Keynes, UK

**Keywords:** RNA vaccines, SARS-CoV-2, Infectious diseases

## Abstract

Early in the SARS-CoV-2 pandemic concerns were raised regarding infection of new animal hosts and the effect on viral epidemiology. Infection of other animals could be detrimental by causing clinical disease, allowing further mutations, and bares the risk for the establishment of a non-human reservoir. Cats were the first reported animals susceptible to natural and experimental infection with SARS-CoV-2. Given the concerns these findings raised, and the close contact between humans and cats, we aimed to develop a vaccine candidate that could reduce SARS-CoV-2 infection and in addition to prevent spread among cats. Here we report that a Replicon Particle (RP) vaccine based on Venezuelan equine encephalitis virus, known to be safe and efficacious in a variety of animal species, could induce neutralizing antibody responses in guinea pigs and cats. The design of the SARS-CoV-2 spike immunogen was critical in developing a strong neutralizing antibody response. Vaccination of cats was able to induce high neutralizing antibody responses, effective also against the SARS-CoV-2 B.1.1.7 variant. Interestingly, in contrast to control animals, the infectious virus could not be detected in oropharyngeal or nasal swabs of vaccinated cats after SARS-CoV-2 challenge. Correspondingly, the challenged control cats spread the virus to in-contact cats whereas the vaccinated cats did not transmit the virus. The results show that the RP vaccine induces protective immunity preventing SARS-CoV-2 infection and transmission. These data suggest that this RP vaccine could be a multi-species vaccine useful to prevent infection and spread to and between animals should that approach be required.

## Introduction

SARS-CoV-2 is an extremely contagious respiratory coronavirus that emerged in China in late 2019 and has since spread globally causing the on-going coronavirus disease 2019 (COVID-19) pandemic. Coronaviruses are enveloped, single-stranded, non-segmented, positive-sense RNA viruses that encode sixteen non-structural proteins and four structural proteins. The structural Spike (S) protein is the major determinant of host cell tropism by binding to the angiotensin-converting enzyme 2 (ACE2) on cells, a type I integral membrane protein that plays an important role in human vascular health. Using the ACE2 receptor to gain entry to cells in the upper respiratory tract (URT), SARS-CoV-2 infection of humans has manifested itself in a wide range of clinical outcomes, from asymptomatic to very severe respiratory infections which in some situations are complicated by immunological dysfunction causing COVID-19 with over 3,4 million fatalities to date. As ACE2 orthologs that are highly similar to the human ACE2 receptor are also present on the cells of a number of other animals it is important to understand whether those potential hosts can play any role in disease spread. Since the first human infections, it has been shown that cats, dogs, ferrets, hamsters, and mink can be readily infected either in laboratory studies or via natural transmission^1–5^. The role these susceptible animals play in the human epidemiology is unclear, though two-way transmission between mink and humans has been demonstrated leading to the culling of all animals in mink farms from Denmark and the Netherlands^3^. It is therefore of utmost importance to understand the role of animals in the spread of this virus, especially with regard to their potential to act as a viral reservoir and to develop important viral variants which thereby influence the overall epidemiology.

The importance of cats in the epidemiology of COVID-19 has yet to be fully established, though there are a significant number of reports of cats testing positive for SARS-CoV-2, mostly in association with human infections in the same household. The first published report demonstrating that cats could be experimentally infected also showed virus transmission to in-contact cats^1^. Whilst the infected cats did not demonstrate overt clinical disease, significant respiratory lesions were detected post-mortem especially in younger cats. In subsequent experimental trials, no clinical disease was observed in challenged cats, but prolonged shed of virus and spread to contact cats was again detected ^45^. In addition to these experimental infection studies, there have been numerous reports of domestic cats testing positive for SARS-CoV-2, with less than a quarter showing signs of disease and no severe presentations as reported in humans^6^. Although a number of these cases were associated with the presence of a confirmed SARS-CoV-2 infected owner, this was not always the case^7^, and natural infection between domestic cats has not been ruled out. Serological surveys of cats in China^7^, USA^8^, France^9^, and Italy^10^ have demonstrated that a high proportion of cats tested positive for SARS-CoV-2, including feral animals with no known history of ownership. Although concerns regarding feline infections have significantly reduced, the initial reports that a large number of cats were being abandoned by owners^11^ led to key opinion leaders releasing statements regarding the low risk of human infection from cats^6^. Furthermore, owners testing positive for SARS-CoV-2 have been advised to distance themselves from their cats in an attempt to prevent transmission, and SARS-CoV-2 infection of cats is now reportable to the World Organization for Animal Health (historically, OIE)^12^. Recently, with the rise of new variants, there are also reports that these variants may have altered host tropism^13^ and possibly different pathogenesis^14^. For these reasons it is important to further study the epidemiology of SARS-CoV-2 in cats, and whether the possibility exists of the feline population becoming a natural reservoir for the virus.

A large number of human vaccines are now in development against SARS-CoV-2, with more than ten approved for use in various regions globally under an Emergency Use Authorization (EUA). The types of vaccine include adjuvanted expressed SARS-CoV-2 S protein, adjuvanted whole SARS-CoV-2 virus vaccines, mRNA encoding SARS-CoV-2 S, and recombinant viral vector vaccines expressing the SARS-CoV-2 S^15^. Early reports indicate that these vaccines have good safety profiles and have greatly reduced both the number and severity of infections. Other important considerations for the long term success of these vaccines include the immunological correlates of the protection induced, the vaccination scheme required to induce an appropriate duration of immunity, protection against variant strains, the cost and production scale of these vaccines required for the global population as well as storage and transportation temperatures, potential rare side effects such as vaccine-induced thrombotic thrombocytopenia^16^ and whether any antibody-dependent enhancement is detected, as has been seen on rare occasions with other coronavirus vaccine candidates^17^. The use of coronavirus vaccines in the veterinary industry is well established with a variety of vaccines against infectious bronchitis virus (IBV), bovine coronavirus (BCV), porcine epidemic diarrhoea virus (PEDV), feline infectious peritonitis (FIP), and canine coronavirus (CCV) being used broadly for many decades. As an interesting parallel to SARS-CoV-2 infection, IBV is transmitted via the respiratory route, initially causing an upper respiratory infection in chickens followed by the systemic disease which, depending on the strain, can involve the kidney, reproductive organs, or the intestinal tract. IBV has evolved into an enormous number of variant strains globally and it is important to note that many different serotypes of IBV are present^18^. These serotypes are sufficiently antigenically distinct that most require unique serotype-specific vaccines to control the disease.

The licensure of IBV vaccines requires not only the demonstration of protection from clinical disease but also a highly significant reduction of virus replication in the trachea. Given its respiratory route of transmission and the requirements to significantly reduce virus present in the respiratory tract, the most effective vaccines are live attenuated viruses, which are delivered by mucosal application, either by spray or in drinking water. Local delivery of live attenuated IBV vaccines induces relatively short-lived local mucosal IgA neutralizing antibody responses in addition to systemic IgA and IgG antibody responses^19–21^. However, the complete mechanism of protection is unclear and is likely to involve cell-mediated immunity specific for other proteins besides the S. In longer-lived birds, responses are boosted by the parenteral delivery of adjuvanted inactivated whole virus vaccines, which extends the duration of immunity significantly and is likely to boost the immune response that has been primed by the mucosally delivered vaccine. Interestingly, the induction of virus-neutralizing serological antibodies against the S protein by parenteral vaccination routes has previously been shown to provide a low level of protection against respiratory IBV challenge^22–24^. It is therefore of particular interest to determine how well the human SARS-CoV-2 vaccine candidates, most of which are designed to be administered parenterally, are able to control upper respiratory tract infection and virus spread, in addition to preventing clinical disease. Initial indications are that these vaccines are successfully reducing human to human spread, though the mechanisms involved require further investigation.

In the controlled experiments in felines, it has been shown that the SARS-CoV-2 virus can readily infect cats and although in most cases no or only a mild disease was detected, the cats can shed the virus for prolonged periods, infecting other cats^4^. Prevention or limitation of virus replication in infected cats, thereby reducing direct contact transmission in cats, would be useful in preventing the establishment of a reservoir in the feline population and also potentially limit the development or selection of viral mutants in these species. In order to investigate the prevention of transmission between cats, we tested whether a vaccine could protect cats from SARS-CoV-2 challenge and also prevent virus spread between cats under controlled conditions.

For this purpose, we utilized an Alphavirus-based Replicon technology derived from the attenuated TC-83 strain of Venezuelan equine encephalitis virus (VEEV) to express the SARS-CoV-2 S protein.

Several other vaccines based on alphavirus replicons have already been designed such as repRNA-CoV2S^25^, LUNAR-COV19^26^, and alphavirus-based DNA-launched self-replicating (DREP)^27^, all based on the SARS-CoV-2 wild-type spike protein and showing promising antibody responses and T cell activation. Additionally, replicon technology has been tested in numerous species (including humans)^28,29^ and the VEEV based replicon has been shown to be safe in cats reducing the clinical effects and shed of Feline Calicivirus, which causes an acute respiratory tract infection (Authors’ unpublished observations). Furthermore, good responses have been detected in chickens, dogs, horses, pigs, and cattle with a variety of antigen targets^28^. The replicon forms the basis of the Sequivity®RNA Particle vaccine platform which is currently licensed in the US for multiple swine applications. In this system, the foreign gene of interest, in this case, SARS-CoV-2 S, is inserted in place of VEEV structural genes generating a self-amplifying RNA capable of expressing the gene of interest upon introduction into cells. The self-amplifying replicon RNA directs the translation of large amounts of protein in transfected cells, reaching levels as high as 15–20% of total cell protein^30^. As the replicon RNA does not contain any of the VEEV structural genes, the RNA is propagation-defective. The replicon RNA can be packaged into replicon particles (RP) by supplying the VEEV structural genes *in trans* in the form of promoter-less capsid and glycoprotein helper RNAs and, when the helper and replicon RNAs are combined and co-transfected into cells, the replicon RNA is efficiently packaged into single-cycle, propagation-defective RP which are used in the vaccine formulation^31^. RP vaccines have been shown to induce both innate and adaptive immune responses including virus neutralizing antibodies and T cell responses^28^. Of significant importance is the fact that this system can be employed very rapidly with materials sufficiently available for deployment of vaccine within weeks.

The structural conformation and cellular localization of the SARS-CoV-2 S protein have been found to be important for the induction of a protective immune response^32–34^. We therefore generated and tested, alongside the wild type variant, five S antigens harboring several types of mutations designed to stabilize the metastable S protein in its prefusion conformation and to increase its cell surface localization. These S antigens were evaluated for protein expression studies in vitro, and were used to generate VEEV RP-based vaccines for testing their immunogenicity in guinea pigs. We further focused on two of the antigen candidates producing either the wild type S antigen (Spike Wt) or an optimized S antigen (Spike Opt), containing the majority of the stabilizing mutations. We compared both antigens—delivered via a plasmid DNA-launched replicon RNA (DREP) vector vaccine or the RP-based vaccine—for their efficacy in eliciting humoral and cellular immune responses. The Spike Opt RP vaccine was selected and evaluated in a cat vaccination-challenge experiment for its ability to protect against SARS-CoV-2 infection and/or prevent transmission to in-contact non-vaccinated cats.

## Results

### SARS-CoV-2 Spike antigen design

**Fig. 1:**
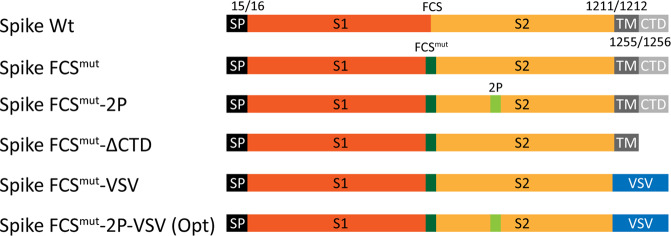
Schematic representation of the SARS-CoV-2 S antigen designs. The position of the wild-type signal peptide (SP), the S1 and S2 subunit, the furin cleavage site (FCS), the transmembrane (TM), and cytoplasmic tail domain (CTD) are indicated. Constructs contain various alterations including FCS mutations (FCS^mut^: R^682^A/R^683^A), proline stabilizing mutations (2P: K^986^P/V^987^P), deletion of the CTD, and replacement of the TM/CTD by that of the vesicular stomatitis virus G protein (VSV).

The recent SARS-CoV-2 vaccine efforts have shown that stabilizing the pre-fusion form of the S protein enhances immunogenicity of the antigen in the mRNA and vector-based vaccines^33,35^. Therefore, we designed five SARS-CoV-2 S antigen forms with combinations of mutations that potentially enhance its stability or cell surface expression, including inactivation of the furin cleavage site (FCS^mut^), the double-proline stabilizing mutations (2P), deletion of the C-terminal domain (∆CTD) and/or the replacement of the CTD and the transmembrane domain (TM) with the counterparts of the G protein of the vesicular stomatitis virus (VSV) (Fig. 1).

### In vitro analysis of modified Spike antigens and immunogenicity study in guinea pigs

**Fig. 2:**
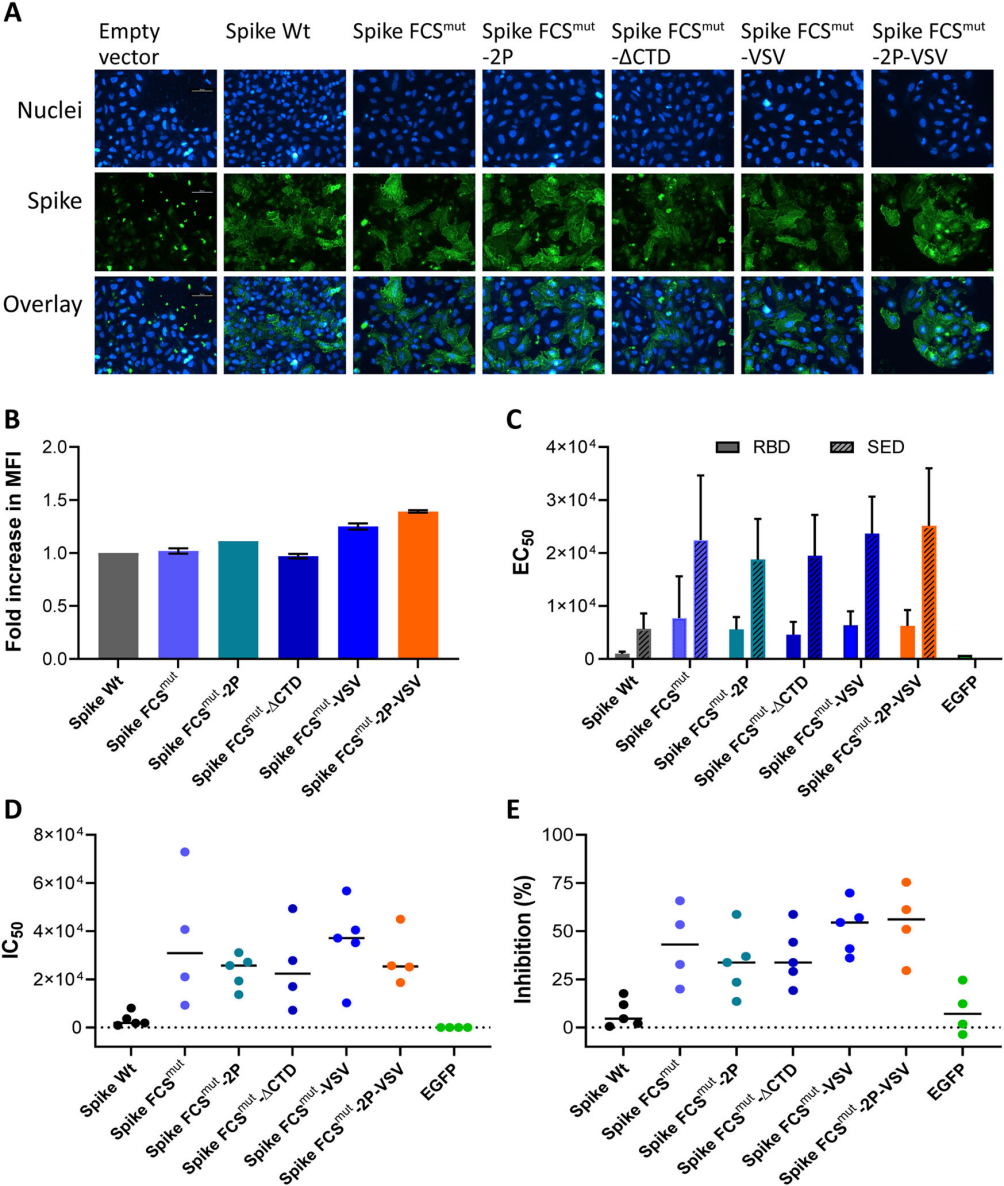
In vitro expression of SARS-CoV-2 S variants and humoral immune responses in vaccinated guinea pigs. **A** Antigen expression levels and localization on the surface of HeLa cells using immunofluorescence microscopy (scale bar 50 µm). Nuclei were stained with Hoechst (blue colour) and S proteins with an anti-RBD targeting antibody (green colour). **B** Quantification of antigen expression levels on the cell surface of plasmid transfected HEK293T cells using FACS by measuring the mean fluorescence intensity (MFI) at 24 h post-transfection. Immunostaining for the spike protein was the same as in (**A**). **C** SARS-CoV-2 S antibody levels in sera of VEEV RP vaccinated guinea pigs, collected at day 56 after vaccination/boost, was measured by ELISA using SARS-CoV-2 S RBD (solid colour) or S ectodomain (pattern) as capturing antigens. Shown are the half-maximal effective concentrations (EC_50_) values of sera collected at the end of the study (expressed as fold dilution). **D** Neutralizing antibody titers against SARS-CoV-2 S pseudotyped VSV determined on Vero E6 cells, and expressed as the half-maximal inhibitory concentrations (IC_50_, fold serum dilution). **E** SARS-CoV-2 surrogate virus neutralization test measuring antibody-mediated blockage of S-ACE2 receptor interaction using 10.000-fold diluted serum samples of guinea pigs, collected at the end of the study.

The surface expression of all six S variants generated from pCAGGS2 plasmids on transfected cells was confirmed by immunofluorescence assay (Fig. 2A) and quantified using flow cytometry (Fig. 2B). A trend towards higher cell surface expression was observed for S forms that included the TM/CTD of VSV-G, especially in combination with the stabilizing 2P mutations (Spike FCS^mut^-VSV and Spike FCS^mut^-VSV).

To assess the immunogenicity of the S variants, we immunized guinea pigs using vaccines based on the VEEV replicon particle (RP) system. Animals were immunized with 1 × 107 VEEV RPs via the intramuscular route at day 0, 21, and 42 of the study, and serum for analysis was collected at day 56. Compared to the wild-type S antigen, inactivation of the FCS (the other five S variants with FCS^mut^) leads to a dramatic increase in ELISA antibody titers against the receptor-binding domain (RBD) or the S ectodomain (SED) (Fig. 2C). Additional mutations introduced into the S protein did not further increase immunogenicity significantly. Neutralizing antibody titers - measured by a commercial SARS-CoV-2 surrogate virus neutralization assay or a pseudovirus neutralization assay—corroborated the ELISA results (Figs. 2D, E). Relative to wild type-S antigen, a trend towards higher neutralization titers were observed for sera of animals immunized with the VEEV RP vaccine producing the S antigens containing a mutated FCS, replacement of the TM/CTD for that of VSV-G in combination with the 2P stabilizing mutations (Spike FCS^mut^-2P-VSV). Based on the cell surface and immunogenicity data we selected this SARS-CoV-2 S form as the lead vaccine antigen, and it will be further referred to as Spike Opt.

### Immunogenicity study of VEEV DREP and VEEV RP vaccine candidates in guinea pigs

**Fig. 3:**
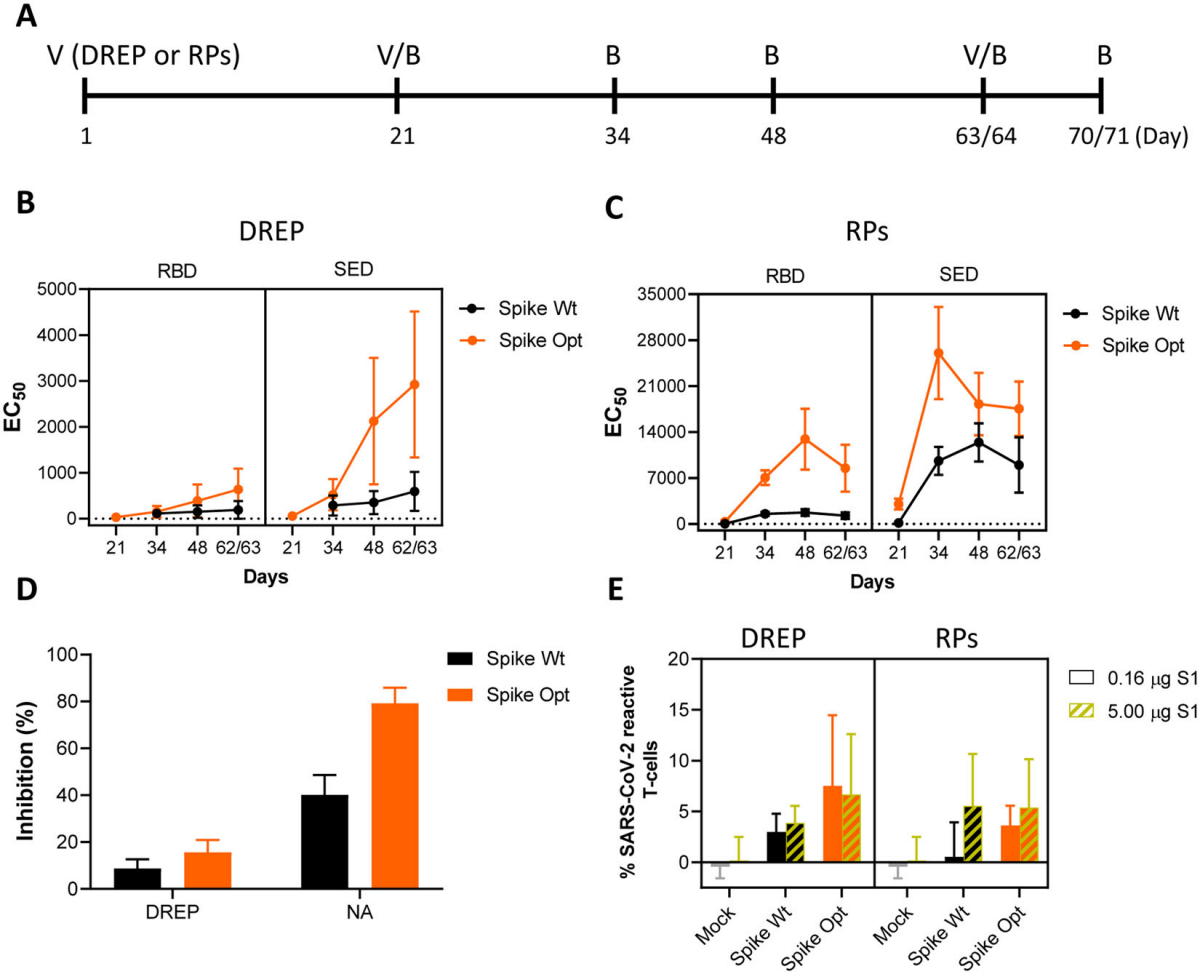
Immunogenicity of vaccine candidates in a guinea pig model. **A** Timeline of animal handlings. V = vaccination with plasmid DNA-launched replicon RNA (DREP) or VEEV replicon particles (RP), B = blood sampling. **B**, **C** Indirect ELISA performed using SARS-CoV-2 S RBD (left) or S ectodomain (right) as capturing antigens. Shown are the EC_50_ values of sera (expressed as fold dilution) from guinea pigs exposed to the wildtype S antigen (Spike Wt) or the optimized S antigen (Spike Opt). **D** SARS-CoV-2 surrogate virus neutralization (VN) assay performed using 1.000-fold diluted serum samples collected after prime-boost vaccination at day 34 and expressed as percent inhibition. **E** Results of lymphocyte stimulation test (LST) using blood collected on day 70/71. Purified SARS-CoV-2 S1 antigen was used to stimulate isolated lymphocytes and proliferation was measured 96 h post-stimulation. Gating-procedure in Supplementary Fig. 1.

Immunogenicity of the Spike Wt and Spike Opt antigens was assessed in a guinea pig model using a plasmid DNA-launched replicon RNA (DREP) based vector vaccine or the VEEV RP vector vaccine, following intramuscularly administration (Fig. 3A). After prime-boost vaccination, all animals showed seroconversion as assessed by both the RBD as well as S ectodomain (SED) indirect ELISA. The DREP vaccine immunizations led to inferior titers as compared to the VEEV RP vaccines (Fig. 3B, C). In line with the previous guinea pig study, the wild-type S induced lower antibody titers compared to the Spike Opt antigen.

Neutralizing antibody titers in sera of immunized animals were quantified using the SARS-CoV-2 surrogate virus neutralization (VN) test. Clearly higher surrogate VN titers were induced by the Spike Opt antigen compared to the Spike Wt antigen in both vaccine platforms (Fig. 3D). The VEEV RP vector platform is known for its efficient induction of both humoral, as well as cellular responses^28^. To assess the level of cellular responses induced by the vaccine candidates delivered as VEEV RPs or DREP, a third immunization was performed and seven days later lymphocytes were isolated for a lymphocyte stimulation test (LST). In contrast to the differences in humoral responses between the two vaccine platforms, DREP immunizations lead to comparable T-cell activation upon stimulation with high concentration (5.0 µg/ml) of SARS-CoV-2 S1 antigen compared to the VEEV RPs (Fig. 3E). Interestingly, at low concentration of SARS-CoV-2 S1 antigen (0.16 µg/ml) the level of reactive T-cells seems to be higher in DREP vaccinated animals compared to VEEV RP vaccinated animals. Concerning the Spike Wt and Spike Opt antigens, a slight increase was observed in levels of SARS-CoV-2 S1 specific T-cell differentiation for the Spike Opt antigens when stimulated with low concentration SARS-CoV-2 S1 antigen, but this difference is no longer noticeable when stimulations were performed with 5 µg of S1 (bars with yellow stripes).

To determine whether the humoral immune response also resulted in mucosal immunity, tracheal swabs were taken at the end of the experiment. Interestingly, surrogate VN titers were detected in the trachea swabs, and the levels correlated with the systemic antibody levels with superior titers for the Spike Opt antigen compared to the Spike Wt antigen (Fig. 3F). These antibody titers suggest that parenteral vaccination could induce protective mucosal immunity.

### Cat vaccination-challenge study

**Fig. 4:**
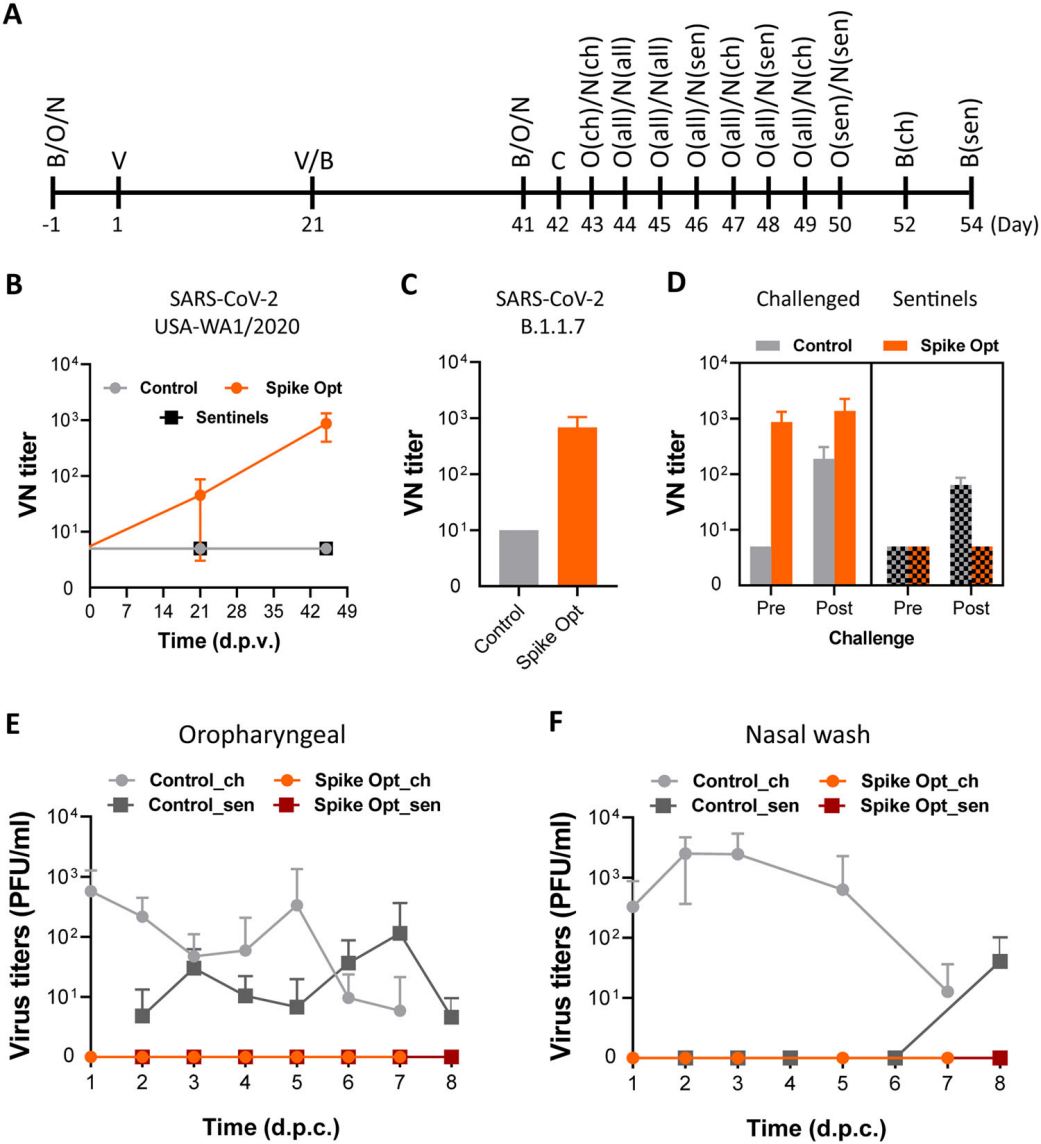
Vaccination-challenge experiment in cats. **A** Timeline of animal handlings. V = vaccination, B = blood sampling, O = oropharyngeal swabs, N = nasal swab, (all) = all animals, (ch) = only challenged animals, (sen) = only sentinel animals. **B** Serum neutralizing antibody titers determined using a SARS-CoV-2 virus neutralization (VN) assay at 21- and 42-days post vaccination (d.p.v.) in the non-vaccinated (Control) or Spike Opt vaccinated cats. **C** Serum neutralizing antibody titers determined using a VN test using the SARS-CoV-2 B.1.1.7 variant virus at 42-days post vaccination in the non-vaccinated (Control) or Spike Opt vaccinated cats. **D** Serum neutralizing antibody titers were determined using a SARS-CoV-2 virus neutralization assay at the day of challenge, 42-days post vaccination and 14 days post challenge and shown for challenged control vaccinated animals (solid grey), non-vaccinated sentinel animal co-housed with control vaccinated animals (grey with pattern), Spike Opt antigen vaccinated animals (solid orange), or non-vaccinated sentinel animals co-housed with Spike Opt antigen vaccinated animals (orange with pattern). SARS-CoV-2 virus titers were measured in oropharyngeal swabs (**E**) or nasal swabs (**F**) at 1−8 days post challenge (d.p.c.) expressed in PFU/ml. All experiments were performed on Vero E6 cells.

To determine vaccine efficacy, cats were either vaccinated with a VEEV RP vaccine producing EGFP (Control), the optimized SARS-CoV-2 S antigen (Spike Opt) or remained non-vaccinated (sentinels). Three weeks post booster vaccination, cats were exposed to a mucosal SARS-CoV-2 challenge using the intranasal and oral routes, and samples were taken as outlined in Fig. 4A.

Following vaccination, no adverse reactions were detected in any of the cats at any time point. The VEEV RP vaccine producing the Spike Opt antigen was able to induce virus-neutralizing antibody titers in all cats after a single vaccination, which was boosted after the second vaccination and maintained levels until the challenge 3.5 weeks later (Fig. 4B). The vaccine-induced antibodies were also potently neutralizing the SARS-CoV-2 B.1.1.7 variant (Fig. 4C), a strain that recently has been associated with clinical manifestation in cats^14^. Control and non-vaccinated sentinel animals remained seronegative at all times up until the challenge. Both challenged and sentinel cats did not demonstrate any clinical signs post challenge. However, nine out of ten control challenged cats shed virus orally (Fig. 4E) and nasally (Fig. 4F) one day after the challenge and for at least 3 days during the observation period. These data show that the mucosal SARS-CoV-2 challenge results in efficient virus replication in the respiratory tract. Higher and more consistent virus shed was detected from the nasal washes whereas the oropharyngeal swabs demonstrated a less consistent pattern, the reason for this being unknown. Interestingly, virus shed was also detected from the nasal washes in two of the non-vaccinated sentinels placed with the control animals one day after the challenge. Moreover, all five sentinel animals housed with the challenged control cats shed virus via the oral route for at least two days demonstrating the efficient spread of the virus from control challenged to sentinel animals (Fig. 4E).

None of the vaccinated cats shed any detectable virus orally (Fig. 4E) or nasally (Fig. 4F) at any time point after the challenge. The results suggest that the vaccine may have prevented infection. Also, no virus was detected in the non-vaccinated sentinels housed with the vaccinated cats, as would be expected considering the lack of challenge virus replication in the vaccinated cats. Analysis of virus-neutralizing antibody titers post-challenge confirmed the findings that both control challenged and sentinel animals were efficiently infected (Fig. 4D). In contrast, no seroconversion was observed in the sentinel animals housed with the vaccinated cats. Thus, the VEEV RP vaccine producing the Spike Opt antigen appears to induce protective immunity and prevent transmission from infected to naïve cats.

## Discussion

With the ongoing pandemic and reports of naturally occurring SARS-CoV-2 infections of a variety of animal species, it is important to understand the epidemiology of this virus in these animal populations especially with regards to the establishment of potential reservoirs, mutations, and transmission within and to other species. SARS-CoV-2 infections in humans can be transmitted to cats and it has been hypothesized that cat-to-cat transmission of virus can take place in a natural setting^1^. It was previously demonstrated that SARS-CoV could infect and spread between cats^23^, but the complete epidemiological picture of feline infection was not fully understood with the rapid eradication of SARS-CoV from humans before it reached a pandemic situation. The situation with SARS-CoV-2 is different as it has become a global issue with the likelihood of becoming endemic in the human population. Whilst an infected cat is considered a low risk for SARS-CoV-2 transmission to humans, to other cats and other species, considering that infected cats shed virus for prolonged periods which can potentially be aerosolised gives credence to the possibility that cats may play some role in the viral epidemiology either by transmitting the virus onwards, enabling further mutations or acting as a virus reservoir. Although routine vaccination of cats is not proposed, should the epidemiological situation change, the availability of a vaccine that can be rapidly produced, updated, and which reduces or prevents viral replication and transmission between cats and other animals will be useful. Furthermore, a vaccine that could be used in a range of susceptible animal species would be preferable.

We developed a safe and effective VEEV replicon particle-based SARS-CoV-2 vaccine for cats. Our data demonstrate that prime-booster immunization with replicon particles encoding a prefusion stabilized S immunogen induced neutralizing antibody responses in sera and mucosal tissues, and provided full protection against SARS-CoV-2 challenge in all cats.

Based on the immunization studies, the S antigen design in combination with the furin cleavage site mutation (FCS^mut^) appeared most critical to the robust induction of a neutralizing antibody response in both guinea pigs and cats, indicating that this prefusion S stabilizing modification can increase immunogenicity. In the guinea pig experiments, it was interesting to note that intramuscular vaccination induced some level of mucosal antibody titers, which was somewhat surprising and is likely to be a wash over from serological induction. It remains to be established whether these antibodies might contribute to the protective immunity that has been observed for this vaccine candidate in cats.

The optimized S RP vaccine successfully induced a virus-neutralizing antibody response in all vaccinated cats after a single vaccination which was boosted upon a second vaccination. Furthermore, the vaccine was able to prevent infection in all vaccinated cats as demonstrated by the lack of virus re-isolation post-challenge. Although there was a strong induction of a serological response in the cats it was not investigated, as was demonstrated in the guinea pig experiments, whether neutralizing antibody was present in the respiratory tract. Furthermore, we did not examine the role of cell-mediated immunity in the prevention of infection of cats nor were we able to extend the experiments to investigate the duration of the immune response induced. The ability to induce local protection from parenterally administered coronavirus vaccines is not well established and in certain veterinary respiratory coronavirus infections mucosally-applied live attenuated vaccines are used to reduce the viral replication at the site of initial infection. These live vaccines induce a relatively brief period of protection, so they are boosted by inactivated adjuvanted whole virus vaccines to establish longer immunity. The use of inactivated vaccines alone in these veterinary settings is not as effective at protecting the local respiratory tract as the live priming inactivated boost approach^24^. For this reason, it is reassuring that a parenterally administered vaccine did appear to provide respiratory protection in the feline model. Future work would be needed to establish whether a single vaccine dose would also be sufficient to protect the cats from infection. Furthermore, the optimal inoculation schedule has not yet been established for this vaccine nor has the duration of immunity that can be induced and whether the ability to prevent infection and spread persists over this time.

This work demonstrates the utility of the VEEV strain TC-83-based replicon particle vaccine platform (Sequivity^®^). RP vaccines based on VEEV have previously been shown to protect cats against viral diseases including some respiratory protection against clinical signs and virus shed in a feline calicivirus infection model, and in addition have been shown to be effective in multiple species including dogs, horses, pigs, cattle, chickens and ducks^28^, authors’ unpublished observations. Furthermore, VEEV based RP vaccines expressing the S proteins from other coronaviruses have been shown to induce virus-neutralizing antibodies^36^. The advantages of RP-based technology is that vaccines can be rapidly prepared if the gene of interest is known to encode a protective antigen. This vaccine platform is safe-by-design as the RP vaccines undergo a non-productive cycle of replication in which replicon RNA but no virus is replicated and no adjuvants are required^31^.

Thus far Rhesus macaques, hamsters, and ferrets have been utilized as natural animal models for SARS-CoV-2. In these animals, infection is usually asymptomatic or induces mild clinical disease. As SARS-CoV-2 also induces asymptomatic infections in cats, this species may also provide a means to study virus transmission, especially via aerosols and vaccine design aimed at preventing initial infection in the respiratory tract. In some respects, infection of cats may mimic the majority of human infections which are asymptomatic.

This work demonstrates that a VEEV replicon-based vaccine expressing the stabilized SARS-CoV-2 S protein was able to induce high levels of virus-neutralizing antibodies in serum of vaccinated cats and that the induced response was able to prevent infection of the upper respiratory tract, thereby preventing onward transmission to other cats.

## Methods

### Animals and husbandry

Female SPF guinea pigs (Dunkin Hartley) were obtained from Envigo at a minimum weight of 350 g, randomly allocated to experimental groups, and individually marked using colour-coded tags. Baseline clinical observations were documented throughout the study period. Domestic short hair male and female SPF cats were obtained from Marshall BioResources (Waverly, NY), identified by microchip, and randomly allocated to experimental groups. Baseline clinical observations including body temperatures were documented throughout the study period.

### Generation of SARS-CoV-2 Spike antigen designs and replicon particle (RP) vaccines

The SARS-CoV-2 S gene (strain 2019-nCoV/USA-WI1/2020, GenBank accession MT039887) was used for generating several S protein antigen designs possessing the R^682^A/R^683^A (FCS^mut^), K^986^P/V^987^P (2P) substitutions, deletion of the C-terminal domain (ΔCTD, residues 1256−1273), replacement of the S transmembrane and C-terminal domains (residues 1212−1273) by that of the VSV G glycoprotein (residues 463−511, GenBank accession YP_009505325). The original SARS-CoV-2 S gene (wt) or S genes encoding these alterations were cloned in pCAGGS2 vectors and used for transient expression in HeLa and HEK293T cells.

The VEEV replicon vectors used to produce either the SARS-CoV-2 Spike Wt gene or the other variants were constructed as previously described^37^ with the following modifications. The TC-83-derived replicon vector “pVEK” was digested with restriction enzymes *AscI* and *PacI* to create the vector “pVHV”.

The Spike Wt gene sequence and the Spike Opt derivative possessing the FCS^mut^-2P-VSV substitutions were codon-optimized for expression in cat and synthesized with flanking *AscI* and *PacI* sites (ATUM, Newark, CA). The synthetic genes and pVHV vector were each digested with *AscI* and *PacI* enzymes and ligated to create vectors “pVHV-SARS-CoV-2-Spike Wt” and “pVHV-SARS-CoV-2-Spike Opt”. Plasmid batches were sequenced to confirm the correct vector and insert identities.

Production of TC-83 RNA replicon particles (RP) was conducted similarly to methods previously described^38^. Briefly, pVHV-SARS-CoV-2-SpikeWt and pVHV-SARS-CoV-2-SpikeOpt replicon vector DNA and helper DNA plasmids were linearized with *NotI* restriction enzyme prior to in vitro transcription using RiboMAX™ Express T7 RNA polymerase and cap analogue (Promega, Madison, WI). Importantly, the helper RNAs used in the production lack the VEE subgenomic promoter sequence, as previously described^31^. Purified RNA for the replicon and helper components were combined and mixed with a suspension of Vero cells, electroporated in 4 mm cuvettes, and returned to serum-free culture media. Following overnight incubation, VEEV replicon particles were purified from the cells and media by passing the suspension through a depth filter, washing with phosphate-buffered saline containing 5% sucrose (w/v), and finally eluting the retained replicon particles (RP) with 400 mM NaCl + 5% sucrose (w/v) buffer or 200 mM Na_2_SO_4_ + 5% sucrose (w/v) buffer. Eluted RP were passed through a 0.22 μm membrane filter and dispensed into aliquots for storage prior to assay and lyophilisation. A control vaccine was also prepared expressing the green fluorescent protein.

The titers of functional RP-S vaccines were determined by immunofluorescence assay on infected Vero cell monolayers following lyophilisation in a stabilizer containing sucrose, NZ Amine, and DMEM and storage at 2−8 °C. Briefly, the vaccine was serially diluted and added to a Vero cell monolayer culture in 96-well plates and incubated at 37 °C for 18−24 h. After incubation, the cells were fixed and stained with the primary antibody (anti-VEEV nsp2 monoclonal antibody) followed by a FITC conjugated anti-murine IgG secondary antibody. RNA particles were quantified by counting all positive, fluorescent stained cells in 2 wells per dilution using the Biotek® Cytation™ 5 Imaging Reader.

The placebo vaccine consisted of RNA Particles expressing the green fluorescent protein (GFP) assayed, lyophilized, and stored at 2−8 °C as described above. Following use, each of the test vaccines were titrated to confirm the vaccination dose.

### Generation of plasmid DNA vaccines

For both the SARS-CoV-2 Spike Wt and SARS-CoV-2 Spike Opt genes a plasmid DNA-based vaccine was constructed. The VEEV TC-83 replicase followed by the SARS-CoV-2 S genes were cloned from the pVHV vector into the pVAX vector downstream of a CMV promoter that drives RNA transcription in the eukaryotic host. Purified pVAX-CMV-VEEV-Rep-SARS-CoV-2-Spike DNA was formulated into Invivofectamine 3.0 Reagent from ThermoFisher according to the manufacturer’s instructions. Formulated plasmid DNA was diluted down to 125 µg/ml in PBS and used to vaccinate guinea pigs.

### Immunofluorescence assay

HeLa cells were seeded in Dulbecco’s modified essential medium (DMEM, Lonza), supplemented with 10% fetal calf serum (FCS, Bodinco) and 100 U/ml penicillin and 100 μg/ml streptomycin (PS, Lonza), at a density of 40000 cells/cm^2^ in 24-well clusters containing glass slides (1 cm diameter). The following day, cells were transfected with 625 ng pCAGGS2 plasmid DNA using polyethyleneimine (Polysciences Inc.) at a DNA:PEI ratio of 1:10. The transfection mixes were prepared in OptiMEM (Lonza), vortexed for 15 s, and then incubated at room temperature for 20 min. Afterwards, 50 µL mix was added per well and the medium was replaced after 7 h of incubation with cells. At 24 h post transfection 50 µL of culture medium containing DAPI (final dilution per well 1:4000) was added in each well and incubated for 15–30 min, after which medium was removed, monolayers were washed one time with DPBS (1× DPBS without Calcium and Magnesium, Lonza) followed by fixing with 3% PFA. After fixing for 1 h cells were washed again with DPBS and blocked for 1 h in 3% BSA (blocking solution). Afterwards, the glass slides were incubated for 1 h at RT with anti-SARS CoV2 S human mAb (targeting the RBD), diluted to 10 µg/mL in blocking buffer. Afterwards, three washing steps of 5 min were performed with 0.05% Tween 20 solution, and the secondary antibody (Goat anti-human IgG, Alexa488, Molecular probes) was added at a 1:400 dilution in blocking buffer. After another 1 h incubation cells were washed again 3 times with 0.05% Tween 20 solution and one time with DPBS. Slides were mounted using 10 µL FluorProtect reagent (Millipore) and stored at room temperature overnight before images were collected with the Olympus BX60 fluorescence microscope.

### FACS analysis

HEK293T cells were cultured in DMEM/10%FCS/PS and seeded at a density of 1 × 10^5^ cells/cm^2^ in six-well clusters. The following day, cells were transfected with 2.5 µg pCAGGS2 plasmid DNA using polyethyleneimine (Polysciences Inc.) at a DNA:PEI ratio of 1:10. The transfection mixes were prepared in OptiMEM (Lonza), vortexed for 15 s, and then incubated at room temperature for 20 min. Afterwards, 200 µl mix was added per well and the medium was replaced after 7 h of incubation with the cells. At 24 h post transfection monolayers were washed one time with DPBS (1× DPBS without Calcium and Magnesium, Lonza) and cells were dissociated by adding 0.32 ml TrypLE (trypsin replacement reagent, Gibco) for 3–5 min at room temperature. Next, cells were mixed by pipetting DMEM (up to 1 ml) and 10 µl suspension was used for counting (Invitrogen, Countess II), while the rest was pelleted by centrifugation for 5 min/1000 rpm. The medium was removed and cells were fixed with 3% PFA for 20 min, on ice. After fixing, cells were pelleted (5 min/2500 rpm/4 °C), permeabilized (or not) for 20 min on ice with 0.5% saponin, and blocked for 1 h in 3% BSA (blocking solution) on ice. Approx. 4 × 10^5^ cells were further used for analysis, from each sample, in duplicate. Blocked cells were moved to round-bottom 96-well clusters, pelleted, and incubated with the primary antibody (human MAbs 47D11 or CR3022), diluted to 10 µg/ml in blocking buffer. Afterwards, three washing steps of 5 min were performed with 0.05% Tween20 solution, and the secondary antibody (Goat anti-human IgG, Alexa488, Molecular probes) was added at a 1:400 dilution in blocking buffer. After 1 h incubation cells were washed again 3 times with 0.05% Tween 20 solution and resuspended in FACS buffer (2% BSA, 5 mM EDTA, 0.02% NaN3), before analysis with the CytoFLEX LX (Beckman Coulter). Results were analyzed with FlowJo v.9 software.

### SARS-CoV-2 Spike pseudotyped-VSV neutralization assay

The method has been previously described^39^. Briefly, at 24 h after seeding, VeroE6 cells were infected with the virus which had been pre-mixed for 1 h with a series of sera dilutions (starting 1:100). At 20 h p.i., the inoculum is removed, cells are washed and lysed in Passive lysis buffer (Promega). The readout was performed with the Berthold Centro LB 960 luminometer, and was based on the activity of firefly luciferase, in the presence of the D-luciferin substrate (Promega).

### Guinea pig immunization

Study 1: SPF guinea pigs with a weight of 350−500 g were randomly divided over the GFP control group and the six vaccine groups (*n* = 5 per group). One week after placement, animals received a prime vaccination of 1 × 10^7^ RP dose intramuscularly (0.3 ml in the thigh or rump). Three and six weeks after prime vaccination animals received a booster vaccination of 1 × 10^7^ RP dose intramuscular. Terminal blood was taken 2 weeks after the last booster vaccination (day 56) using cardiac-puncture and serum was used to determine systemic antibody titers.

Study 2: SPF guinea pigs with a minimum weight of 350 g were randomly divided over the non-vaccinated control group, RP-Spike Wt vaccine group, and RP-Spike Opt vaccine group (*n* = 6 per group). One week after placement, animals remained either non-vaccinated or received a prime vaccination of 1 × 10^7^ RP or 25 µg DREP dose intramuscularly (0.1 ml in each leg muscle). Three weeks after prime vaccination animals received a booster vaccination. Six weeks after the booster vaccination animals received a second booster vaccination and 7 days later animals were sacrificed. Terminal blood was taken for LST and trachea was carefully dissected without causing bleedings. Mucus was taken from the inside of the trachea using a swab, taken up in 1 ml of phosphate-buffered saline, and used to determine mucosal antibody titers. At the day of booster vaccination, and with 2-week interval until 6 weeks after boost vaccination, clotted blood was taken using cardiac-puncture, and serum was used to determine systemic antibody titers.

### SARS-CoV-2 surrogate VN assay

The SARS-CoV-2 Surrogate Virus Neutralization Test Kit from GenScript (REF: L00847) was used according to the manufacturer’s instructions. Briefly, sera were diluted in sample dilution buffer, mixed 1:1 with HRP-RBD, and incubated 30 min at 37 °C. Next, samples were put in a 96-well plate containing ACE2 receptor coated on the surface and incubated 15 min at 37 °C. Unbound HRP-RBD was washed away and the remaining HRP was visualized using TMB substrate and measured at OD_450_.

### ELISA assay

Purified SARS-CoV-2 S receptor-binding domain (RBD) and S ectodomain (SED), produced as previously described^40^ were diluted in DPBS (without Ca and Mg, Lonza, 17-512F) and coated onto 96-well plates (MaxiSorp—ThermoFisher or High binding—Greiner Bio-one) using 10 nM (10 pmols/ml), and incubated overnight at 4 °C. Next morning plates were washed with an ELISA plate washer (ImmunoWash 1575, BioRad) using 0.25 ml wash solution/well (DPBS, 0.05% Tween 20) three times, then blocked with 250 µl blocking solution (5% milk—Protifar, Nutricia, 0.1% Tween 20 in DPBS) for 2 h at RT (room temperature). Afterwards, the blocking solution was discarded, four-fold serial dilutions of the sera (prepared in blocking solution, in duplicates or triplicates) were added to the corresponding wells and incubated for 1 h at RT. Each plate contained positive control (guinea pig sera diluted to obtain an OD450 of ~2) and negative control wells. Plates were washed again 3 times before being incubated with the HRP-containing antibody—Goat anti-Guinea pig (IgG-HRPO, Jackson Lab 106-035-003, 1:8000) for 1 h at RT. The last washing steps were performed, followed by incubation for 10 min at RT with 100 µl/well Super Sensitive TMB (Surmodics, TMBS-1000-01). Reactions were stopped by adding 100 µl/well of 12.5% H_2_SO_4_ (Millipore, 1.00716.1000). Absorbance at 450 nm was measured within 30 min with an ELx808 Biotek plate reader.

### T-cell stimulation test (LST)

Blood was collected and lymphocytes were isolated using Sepmate tubes (Stemcell) containing Histopaque 1083 according to the manufacturer’s instructions. Briefly, K3-EDTA blood was diluted 1:2 in RPMI-1640 medium and pelleted for 10 min at 1.200 × *g*. Cells in the top layer of the tubes were collected, put in a clean tube containing RPMI-1640, and pellet for 7 min at 400 x *g*. Cells were washed once with RPMI-1640 medium and pelleted for 7 min at 400 × *g*. Cell densities were counted and 1 × 10^7^ cells were stained with CFSE for 20 min at 37 °C. Cells were washed once with RPMI-1640 and from each animal 5 × 10^5^ cells were stimulated with either medium, ConA (10 µg/ml), or purified SARS-CoV-2 S1 antigen (5.0 or 0.16 µg/ml) in duplicate. Four days after stimulation, cell proliferation based on CFSE stain reduction was measured using the FACS Verse (Beckton Dickinson).

### SARS-CoV-2 virus culturing

SARS-CoV-2 strain USA-WA1/2020 (GenBank: QHO60594.1) was isolated from an oropharyngeal swab from a patient with a respiratory illness who had returned from travel to an affected region of China and developed the clinical disease (COVID-19) in January 2020 in Washington, USA. The virus was propagated for one passage on VeroE6 cells. To determine the virus titer, serial dilutions of virus were made on VeroE6 cells and plaque-forming units quantified by counterstaining with a secondary overlay containing Neutral Red at 24 h and visualization after 48 h of incubation.

### Feline serology

Serological responses to SARS-CoV-2 were studied using an invitro plaque reduction neutralization test (PRNT). Briefly, serum was inactivated at 56 °C for 30 min, serial dilutions of cat serum were prepared and incubated with 100 PFU of SARS-CoV-2 for 1 h at 37 °C. The virus serum mixtures were then plated onto Vero cells and the number of plaques read by counterstaining with a secondary overlay containing Neutral Red at 24 h and visualization after 48 h. Antibody titers were determined as the reciprocal of the highest dilution in which ≥90% of virus was neutralized.

### Cat vaccination/challenge experiment

Two groups of ten 11-week-old SPF cats were formed and housed separately; one group was vaccinated with 5 × 10^7^ RP-Spike Opt by the subcutaneous route (0.5 ml per dose) with the other group receiving the same dose of RP-GFP. After three weeks each group received the same treatment. Twenty-five days following the second vaccination the cats were exposed to a mucosal challenge as previously described^4^ though using both the intranasal and oral routes with 3.1 × 10^5^ PFU of SARS-CoV-2 under light sedation. Two additional groups of five SPF cats that were neither vaccinated nor challenged were used as sentinels by co-housing with each vaccine group 1 day post-challenge. All animals were observed daily for 10 days following challenge for clinical signs indicative of SARS-CoV-2 infection. Clinical signs checked included depression, dyspnea, nasal discharge, ocular discharge, cough, conjunctivitis, and/or sneezing. Body temperatures were recorded on study days 1−11 post-challenge/post-mingling. Oropharyngeal swabs for virus isolation were collected from the challenged cats on study days 1−7 post-challenge, the swabs were placed in Tris-buffered MEM containing 1% bovine serum albumin containing gentamycin, penicillin, streptomycin, and amphotericin B (BA-1 media). To assess contact spread swabs were also collected from the contact sentinels into transport media on study days 2–8 post-challenge. The samples were frozen at −80 °C until testing. Nasal wash samples for virus isolation were collected on days 1, 2, 3, 5, and 7 post-challenge as previously described^4^ by instilling 1 ml of BA-1 media into the nares of cats and collecting nasal discharge in a petri dish. To assess contact nasal washes were also collected from the contact sentinels on days 2, 3, 4, 6, and 8 post-challenge. The samples were frozen at −80 °C until testing. Blood samples were taken for sera prior to and 3 weeks post-primary vaccination. In addition, blood samples were taken prior to and 14 days post-challenge. All oropharyngeal swabs and nasal washes were tested for virus re-isolation as previously described^4^. Confluent monolayers of Vero E6 cells in 6-well plates were washed once with PBS and seeded with 100 μl of serial ten-fold dilutions of swab/wash samples, incubated at 37 °C for 1 h then overlaid with 0.5% agarose in MEM containing 2% FBS. A second overlay containing neutral red dye was added 24 h later and plaques counted at 48 h. Viral titers were recorded as log_10_ PFU/ml.

### Statistical analyses

A two-tailed T-test was used to compare serological responses. *P*-values of less than 0.05 were considered to be significant.

### Ethics section

Studies in cats were approved by the Institutional Animal Care and Use Committee (IACUC) for Merck under permit E-2020-67 and for CSU under permit 1108. Guinea pig studies were approved by IACUC for Merck under permit 20-26 and by the Dutch Central Commission for Animal experimentation (CCD) under permit AVD2210020209944-appendix 1.

### Reporting summary

Further information on research design is available in the Nature Research Reporting Summary linked to this article.

## Supplementary information

Reporting Summary

Supplementary Information

## Data Availability

The datasets generated and/or analyzed during the current study are available from the corresponding author on reasonable request.
