# Supplementary material for: An alphavirus replicon-based vaccine expressing a stabilized Spike antigen induces protective immunity and prevents transmission of SARS-CoV-2 between cats
Source: NPJ Vaccines. 2021 Oct 20;6:122. doi: 10.1038/s41541-021-00390-9 (PMC8528862; doi:10.1038/s41541-021-00390-9)
Supplement: Supplementary file 1 — Reporting Summary [file 41541_2021_390_MOESM1_ESM.pdf]

nature portfolio

Corresponding author(s): Dr. M.A. Langereis

Last updated by author(s): 08-SEP-2021

Reporting Summary

Nature Portfolio wishes to improve the reproducibility of the work that we publish. This form provides structure for consistency and transparency in reporting. For further information on Nature Portfolio policies, see our Editorial Policies and the Editorial Policy Checklist.

Statistics

For all statistical analyses, confirm that the following items are present in the figure legend, table legend, main text, or Methods section.

n/a	Confirmed	
☐	☒	The exact sample size (n) for each experimental group/condition, given as a discrete number and unit of measurement
☐	☒	A statement on whether measurements were taken from distinct samples or whether the same sample was measured repeatedly
☒	☐	The statistical test(s) used AND whether they are one- or two-sided<br>*Only common tests should be described solely by name; describe more complex techniques in the Methods section.*
☒	☐	A description of all covariates tested
☒	☐	A description of any assumptions or corrections, such as tests of normality and adjustment for multiple comparisons
☐	☒	A full description of the statistical parameters including central tendency (e.g. means) or other basic estimates (e.g. regression coefficient) AND variation (e.g. standard deviation) or associated estimates of uncertainty (e.g. confidence intervals)
☒	☐	For null hypothesis testing, the test statistic (e.g. F, t, r) with confidence intervals, effect sizes, degrees of freedom and P value noted<br>*Give P values as exact values whenever suitable.*
☒	☐	For Bayesian analysis, information on the choice of priors and Markov chain Monte Carlo settings
☒	☐	For hierarchical and complex designs, identification of the appropriate level for tests and full reporting of outcomes
☒	☐	Estimates of effect sizes (e.g. Cohen's d, Pearson's r), indicating how they were calculated

Our web collection on statistics for biologists contains articles on many of the points above.

Software and code

Policy information about availability of computer code

Data collection	No software was used
Data analysis	Graphpad Prism 8

For manuscripts utilizing custom algorithms or software that are central to the research but not yet described in published literature, software must be made available to editors and reviewers. We strongly encourage code deposition in a community repository (e.g. GitHub). See the Nature Portfolio guidelines for submitting code & software for further information.

Data

Policy information about availability of data

All manuscripts must include a data availability statement. This statement should provide the following information, where applicable:

- Accession codes, unique identifiers, or web links for publicly available datasets
- A description of any restrictions on data availability
- For clinical datasets or third party data, please ensure that the statement adheres to our policy

The datasets generated and/or analysed during the current study are available from the corresponding author on reasonable request.

Field-specific reporting

Please select the one below that is the best fit for your research. If you are not sure, read the appropriate sections before making your selection.

☒ Life sciences ☐ Behavioural & social sciences ☐ Ecological, evolutionary & environmental sciences

For a reference copy of the document with all sections, see nature.com/documents/nr-reporting-summary-flat.pdf

Life sciences study design

All studies must disclose on these points even when the disclosure is negative.

Sample size	Guinea pig studies were performed with n=5-6 animals per group. Cat study was performed with n=10 animals per group. Previous studies have shown that this amount of animals is enough to show clear differences in serology responses between groups.
Data exclusions	In the guinea pig study some animals had to be euthanized before the end of the study, but no samples were taken. No data was excluded from the results.
Replication	Plasmid DNA tranfection studies have been performed at leat 3 times, represented data are sown in the manuscript. All animal studies have been performed only once.
Randomization	Animals have been randomly allocated to the different experimental groups.
Blinding	Most analysis were performed externally. All samples have been marked with animal numbers, which could not be linked to the different groups.

Reporting for specific materials, systems and methods

We require information from authors about some types of materials, experimental systems and methods used in many studies. Here, indicate whether each material, system or method listed is relevant to your study. If you are not sure if a list item applies to your research, read the appropriate section before selecting a response.

Materials & experimental systems

n/a	Involved in the study	
☐	☒	Antibodies
☐	☒	Eukaryotic cell lines
☒	☐	Palaeontology and archaeology
☐	☒	Animals and other organisms
☒	☐	Human research participants
☒	☐	Clinical data
☒	☐	Dual use research of concern

Methods

n/a	Involved in the study	
☒	☐	ChIP-seq
☐	☒	Flow cytometry
☒	☐	MRI-based neuroimaging

Antibodies

Antibodies used	SARS-CoV-2 MoAb 47D11 or CR3022
Validation	MoAb 47D11: Wang et al., 2020, Nat Comm (doi: 10.1038/s41467-020-16256-y.)<br>CR3022: Tian et al.,, Emerg Microbes Infect. 2020 Feb 17;9(1):382-385. doi: 10.1080/22221751.2020.1729069.

Eukaryotic cell lines

Policy information about cell lines

Cell line source(s)	Vero-E6: ATCC<br>HEK293T: ATCC<br>HeLa: ATCC
Authentication	None of the cells were autenticated
Mycoplasma contamination	All cells were negative for mycoplasma
Commonly misidentified lines (See ICLAC register)	n/a

Animals and other organisms

Policy information about studies involving animals; ARRIVE guidelines recommended for reporting animal research

Laboratory animals	Female SPF guinea pigs (Dunkin Hartley) were obtained from Envigo<br>Domestic short hair male and female SPF cats were obtained from Marshall BioResources (Waverly, NY)
Wild animals	No wild animals used
Field-collected samples	No samples collected in the field
Ethics oversight	Studies in cats were approved by the Institutional Animal Care and Use Committee (IACUC) for Merck under permit E-2020-67 and for CSU under permit 1108. Guinea pig studies were approved by IACUC for Merck under permit 20-26 and by the Dutch Central Commission for Animal experimentation (CCD) under permit AVD2210020209944-appendix 1.

Note that full information on the approval of the study protocol must also be provided in the manuscript.

Flow Cytometry

Plots

Confirm that:

- ☐ The axis labels state the marker and fluorochrome used (e.g. CD4-FITC).
- ☐ The axis scales are clearly visible. Include numbers along axes only for bottom left plot of group (a 'group' is an analysis of identical markers).
- ☐ All plots are contour plots with outliers or pseudocolor plots.
- ☐ A numerical value for number of cells or percentage (with statistics) is provided.

Methodology

Sample preparation	Blood was collected and lymphocytes were isolated using sepmate tubes
Instrument	BD FACSVerse
Software	FlowJo
Cell population abundance	Total lymphocytes were used, no specific staining. Prolofiration was measured using a fluorescent dye.
Gating strategy	Lymphocytes were selected based on FCS/SSC, Single cells were selected using FSC-H/FSC-A, and stimulated cells were measured by decrease in fluorescence signal.

☒ Tick this box to confirm that a figure exemplifying the gating strategy is provided in the Supplementary Information.
