# Supplementary material for: An alphavirus replicon-based vaccine expressing a stabilized Spike antigen induces protective immunity and prevents transmission of SARS-CoV-2 between cats
Source: NPJ Vaccines. 2021 Oct 20;6:122. doi: 10.1038/s41541-021-00390-9 (PMC8528862; doi:10.1038/s41541-021-00390-9)
Supplement: Supplementary file 2 — Supplementary Information [file 41541_2021_390_MOESM2_ESM.pdf]

Supplementary Figure 1

Medium control, Guinea pig 3.4

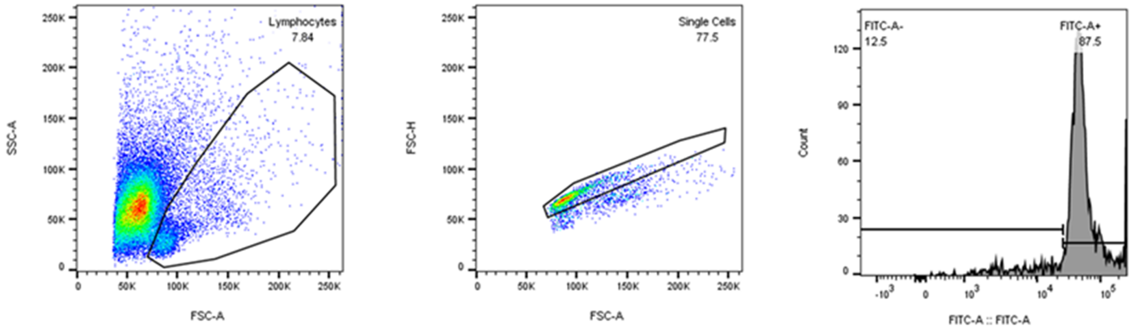

Guinea Pig 3.4, Stimulated with 5 µg/ml SARS-CoV2

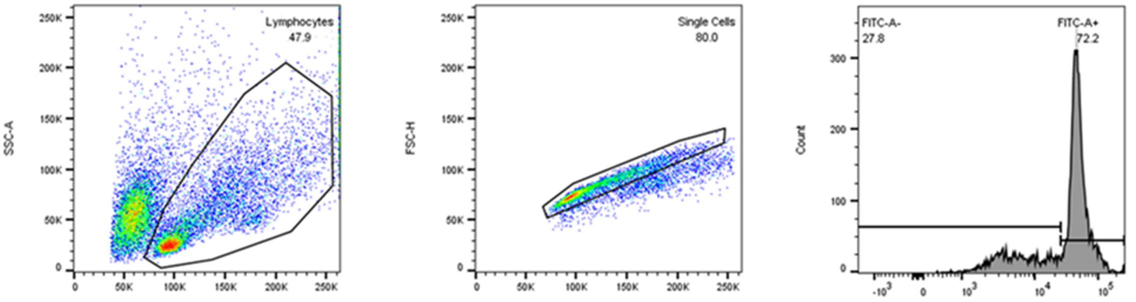

Supplementary Figure 1: Gating procedure used to quantify the level of stimulated lymphocytes in vaccinated guinea pigs. First, lymphocytes were gated based on SSC-A and FSC-A channels. Next, doublets were excluded from the analysis using the FSC-H and FSC-A channels. Finally, the number of proliferated lymphocytes were calculated based on reduced FITC signal gate. Overview of these results are shown in Figure 3.
